# Tissue-specific assessment of oxidative status: Wild boar as a case study

**DOI:** 10.3389/fvets.2023.1089922

**Published:** 2023-03-06

**Authors:** O. Alejandro Aleuy, Diana Gassó, Asta Tvarijonaviciute, David Risco, Waldo Garcia, Pilar Gonçalves, Pedro Fernández-Llario, Gregorio Mentaberre, Roser Velarde, Emmanuel Serrano, Rafaela Cuenca

**Affiliations:** ^1^Department of Biological Sciences, University of Notre Dame, Notre Dame, IN, United States; ^2^Wildlife Ecology and Health Group (WE&H), Departament de Ciència Animal, Escola Tècnica Superior d'Enginyeria Agrària (ETSEA), Universitat de Lleida (UdL), Lleida, Spain; ^3^Interdisciplinary Laboratory of Clinical Analysis (Interlab-UMU), Regional Campus of International Excellence “Campus Mare Nostrum”, University of Murcia, Murcia, Spain; ^4^Innovación en Gestión y Conservación de Ungulados S.L., Cáceres, Spain; ^5^Departamento de Medicina Animal, Faculta de Veterinaria, Universidad de Extremadura, Cáceres, Spain; ^6^Wildlife Health and Ecology Group (WE&H), Servei d' Ecopatologia de Fauna Salvatge (SEFaS), Departament de Medicina i Cirurgia Animals, Facultat de Veterinària, Universitat Autònoma de Barcelona (UAB), Bellaterra, Barcelona, Spain; ^7^Servei d'Hematologia Clínica Veterinaria (SHCV) – Veterinary Clinical Hematology Service, Departament de Medicina i Cirurgia Animals, Facultat de Veterinària, Universitat Autònoma de Barcelona (UAB), Bellaterra, Barcelona, Spain

**Keywords:** glutathione peroxidase, *Mycobacterium tuberculosis*, physiological ecology, p-nitrophenyl esterase activity, *Sus scrofa*, oxidative stress

## Abstract

In recent decades, there has been a fast-growing interest in using biomarkers of oxidative stress (BOS) in conservation programs of many vertebrate species. Biomarkers of oxidative stress can be measured in different biological samples (e.g., body fluids and tissues). However, since comparisons of the same battery of BOS among tissues of the same individual are scarce in the literature, the chosen target tissues regularly rely on arbitrary decisions. Our research aimed to determine if the oxidative status of free-ranging wild boar (*Sus scrofa*) naturally infected with *Mycobacterium* spp (etiological agent of tuberculosis, TB), varies depending on the sample where it was quantified. We compared antioxidant p-nitrophenyl esterase activity (EA), glutathione peroxidase (GPX) concentrations, and total oxidative status (TOS) in serum, lung, spleen, kidney, and muscle of 63 wild boar hunter-harvested in central Spain. Biomarkers of oxidative stress in serum had higher concentrations than in other tissues. The poor agreement between serum and other tissues highlights the importance of running complete BOS assessments in the same fluid or tissue. Further, low concentrations of BOS in tissues of TB-affected individuals were observed, and significant differences between healthy and sick boar were only detected in the serum of individuals developing mild TB and in the muscle of individuals with mild or severe disease status. However, all organs from wild boars affected with mild TB were not in oxidative imbalance compared to healthy control animals, suggesting that wild boars may cope well with TB. Our data indicate that serum and other tissues can be used as BOS in field conservation programs to monitor wildlife population health. Still, context-specific validations are needed to determine the most appropriate samples to use.

## Graphical Abstract

This drawing represents the concept of oxidative stress resulting from an imbalance arising as a consequence of the rate of production of reactive oxygen species exceeding the capacity of the antioxidant defence and repair mechanisms. Oxidative stress then leads to subsequent oxidative damage to biomolecules, tissue dysfunction and disease. Our work is intended to explore how the choice of a biological sample (e.g., muscle, serum, organs) influences oxidative status assessment in wild boar naturally infected with tuberculosis. 
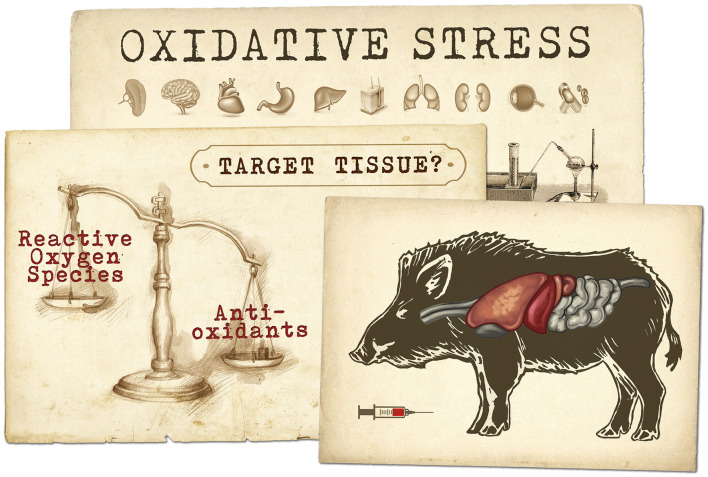

## 1. Introduction

The loss of biodiversity due to anthropogenic disturbances like habitat loss, fragmentation, emerging infectious diseases, and climate change is one of the most critical environmental challenges affecting our planet (1). Assessing the physiological response of animals to these environmental disruptions is an essential component of management, monitoring, and conservation programs in wildlife populations [e.g., (2)]. Biomarkers like stress hormones (glucocorticoids) (3) and immune response indicators (4) have regularly been used for this objective, achieving results with different degrees of robustness [see this review Kalliokoski et al. (5)]. In recent years, there has been a growing interest in incorporating oxidative stress biomarkers (BOS) to assess the physiological response of wildlife to environmental disturbances due to their stability and easy quantification (6, 7). Oxidative stress (OS) is an imbalance between reactive oxygen species (ROS) and antioxidants in living systems. The ROS are unstable molecules (i.e., free radicals) with potential to damage to cell structures, including lipids and membranes, proteins, and DNA (8). There is a rising body of research linking OS to reproduction (9), growth (10), survival (11), immune response (12), and particularly health/disease status (13–15).

Oxidative damage, and in consequence, OS, is generally higher in cells, tissues, and organs suffering from inflammation or local immune response [e.g., erythrocytes, (16); liver (17); intestines (18); kidneys (19) and lungs (20)]. This fact hampers sampling opportunities for researchers working with live animals who prefer to collect sperm (7, 21) or blood serum (22) to avoid animal killing. However, body fluids often fail to detect oxidative damage even in sick individuals (6, 23), making any attempt to monitor OS by non-invasive methods more complicated. Ideally, the choice of specific BOS and the biological samples to properly evaluate OS should be determined on a system-specific basis, considering the particular aims of the study, the experimental design (e.g., access to samples), and the clinical relevance in the selected subjects (24). Unfortunately, very little information is available to inform these decisions, specifically about the agreement between BOS measurements obtained from different tissues (25, 26). Consequently, there is a growing need for experimental and observational studies to evaluate the suitability of specific BOS and biological samples to assess vertebrates' oxidant and antioxidant status. These studies are of significant importance when investigating animals facing conservation issues due to reduced sample availability and in the case of animals infected with zoonotic pathogens, as biosafety measures need to be put in place before sampling occurs.

In this study, we investigated how BOS collected from various body tissues relates to OS in animals with different health statuses. To do this, we compared the concentrations of BOS, antioxidants, and the specific oxidative imbalance in serum, lung, spleen, kidney, and muscle of wild boar (*Sus scrofa*) hunter-harvested from populations with endemic tuberculosis. The Graphical Abstract at the top of this page summarizes the aims of our work. Tuberculosis (TB) is an infective zoonosis caused by *Mycobacterium* spp. This bacteria produces chronic inflammation and systemic OS (27, 28), resulting in lung fibrosis and dysfunction (27–29). Wild boar is the main reservoir of TB in the Mediterranean basin (30), showing differential degrees of resistance to disease progression due to environmental factors (31), genetic characteristics (32), and concomitant infections (33). Boars can develop severe TB with gross lesions in lymph nodes (i.e., mild TB) and the lungs, liver, and spleen (33). A more severe TB implies a more significant bacillary load (34) and lower survival probability (31). First, we compared BOS from different tissues and degrees of TB infection (i.e., negative, mild TB, severe TB). Then, we explored whether BOS measurements are interchangeable among different biological samples from the same individual. Finally, to define the most reliable target tissue to assess OS caused by TB in wild boar, we compared BOS concentrations among different organs and the serum of diseased and healthy animals. Recent work has linked TB progression with OS in the serum of wild boar (35), but, as in most vertebrates, there is no previous information about the most reliable biological sample to assess OS in TB-infected animals. Likewise, no information exists regarding the oxidant-antioxidant imbalance in animals showing severe TB.

## 2. Materials and methods

### 2.1. Wild boar and study area

A total of 63 wild boar (50 females and 13 males) aged from 5 to 60 months were hunter-harvested in a private 2,000 ha hunting state in Toledo (central Spain; 39°55′ 15.55″N, 5 °10′ 32.33″ E). For each hunted animal, the objective was to cause the least animal welfare harm and the least impact in stress possible. This was done by attempting shoots that cause a short flight distance (i.e., the actual distance traveled by an animal from where it was initially struck by the first bullet to where it fell recumbent, immobile, and regarded as unconscious) and dead. The vegetation in this area is a Mediterranean forest dominated by scattered holm oaks (*Quercus ilex*) and Mediterranean shrubs, primarily *Cistus ladanifer, Erica* spp., and *Genista anglica*. Wild boar density in the study area was about 40 boars per 100 ha (unpublished data). Supplemental feeding (Jabalí Familia, Mercoguadiana, S.A., Spain) is regularly provided during summer when natural resources are scarce. Boars were legally hunted in their habitat by authorized hunters within the framework of an annual hunting plan approved by the regional authority in charge of livestock and wildlife management. No approvals were needed from an animal ethic committee since the wild boars were not sacrificed for research purposes.

### 2.2. Tissue samples

After animal culling, sex was determined through a visual inspection of the genitalia area, while age was assessed by dental biometry (36). The positivity of wild boars to *M. bovis* was determined by microbiological cultures from intact submandibular or retropharyngeal lymph nodes and a piece of a caudal lung lobe following the methods described in Gassó et al. (35). Animals were classified into three groups: (i) TB-free boars (negative culture and no signs of TB lesions), (ii) positive culture and localized gross lesions (mild TB), and (iii) positive culture and disseminated gross lesions (severe TB) (33, 37).

Within the first 5 h after culling, blood, lung, spleen, kidney, and muscle samples were collected, placed in cold boxes at 4°C, transported to the laboratory, and stored at −80°C. Blood was collected from the cavernous sinus (38) in plain tubes, and the tubes were centrifugated at 3,500 rpm (1068 g) for 10 min to obtain serum. Before analysis, lung, spleen, kidney, and muscle samples were processed according to Renerre et al. (39). In brief, tissue samples were homogenized in a 50 mM phosphate buffer, pH 7.0, and centrifuged at 1,000 g for 15 min at 4°C. The supernatant was transferred to Eppendorf tubes and used for analyses. Highly hemolytic samples were identified by visual inspection and eliminated from the study due to their potential interference with the analytical methods (40).

### 2.3. Oxidative stress biomarkers analysis

We used the antioxidant p-nitrophenyl esterase activity (EA), glutathione peroxidase (GPX) concentrations, and total oxidative status (TOS) analysis to describe the overall picture of vertebrates' oxidant (TOS) and antioxidant (EA, GPX) balance. The EA (IU/mL) was analyzed by measuring the hydrolysis of p-nitrophenyl acetate to p-nitrophenol as described elsewhere (41). The reaction was monitored at 405 nm. The non-enzymatic hydrolysis of phenylacetate, based on the hydrolysis rate in the absence of a sample, was subtracted from the total hydrolysis rate. The molar extinction coefficient used to calculate the rate of hydrolysis was 14,000 M^−1^ cm^−1^ (41). When EA is measured in serum using this method, it is named paraoxonase 1 (PON1, also in IU/ml) because, in serum, this enzyme has shown activity when p-nitrophenyl-acetate is used as substrate (42). In other tissues, it is not well characterized yet; thus, it is generally called EA. This enzyme has been considered in our study because it protects against bacterial infections and destroys oxidized lipids [wild boar stores large amounts of fat in tissues and organs (37)].

The GPX (IU/L) was determined with a commercial kit (Randox Laboratories, UK). The method of GPX activity determination was based on the reaction by which GPX catalyzes glutathione oxidation by cumene hydroperoxide. In the presence of glutathione reductase and NADPH, the oxidized glutathione was immediately converted to the reduced form with concomitant oxidation of NADPH to NADP+, and the corresponding decrease in absorbance at 340 nm was measured. Previous work showed the changes in GPX due to *M. bovis* infection in experimentally infected boars (35).

The TOS (μmol/L) was measured as previously described by Erel (43), with some modifications (44). The method is based on the reaction that the ferric ion makes a colored complex with xylenol orange in an acidic medium. The color intensity, measured at 560 nm using 800 nm as the reference, is related to the total amount of oxidant molecules present in the sample. The assay was calibrated with hydrogen peroxide. The EA, GPX, and TOS analyses were performed in an automatic analyzer (AU 600 automated biochemical analyzer, Olympus, Minneapolis, Minn) to minimize pipetting errors and execution time (44). The intra- and inter-variation of coefficients among all the methods were below 15%, and linearity under the dilution coefficient of variation was close to 1 in all cases.

In the present study, we decided not to correct tissue extracts for the total protein concentration of the sample. Such correction could result in situation-related bias (45) and additional expenses not necessarily contributing to accurate result interpretation. Additionally, we performed a preliminary test with corrected and raw values of the BOS measured in lung tissues. We got identical results to those shown throughout this work (the correlation between corrected and raw pairs was over 0.8 in all cases, results not shown).

### 2.4. Statistical analysis

Descriptive statistics (mean, standard deviation, minimum, and maximum) and Kruskal–Wallis tests were used to compare EA/PON1, GPX, and TOS mean rank concentrations among biological samples. The non-parametric statistic was used due to the skewed distribution shown by our biomarkers. Later, a Bland–Altman (BA) analysis was used to explore the agreement between enzyme concentrations in the lung, spleen, kidney, muscle, and serum samples. This graphical method is the most popular for comparing two measurement techniques (46) using the representation of the mean differences (Y-axis) and magnitude of such measurements (X-axis). The limits of agreement (LoA) at 95% defined as the mean differences ± 1.96 x SD are also estimated and represented. Variables were log-transformed when differences were proportional to means values (47). However, the BA plots do not distinguish between fixed and proportional bias (48). To control this, we explored plots of differences between biomarker values, expressed as percentages of the values on the Y-axis [e.g., (GPX in serum– GPX in Lungs)/mean%], vs. the mean of the two measurements. Finally, a *t*-test based on 1000 bootstrapped replications was used to compare the mean values of our BOS indicators between health and TB-affected boars (mild and severe TB).

Bland-Altman analysis was performed in the “blandr” package version 0.4.3 (49), whereas bootstrapping in the “boot” package version 1.3–20 (50) of the R software 4.2.2 version (51).

## 3. Results

### 3.1. Comparison of BOS values in different tissues

**Figure 1:**
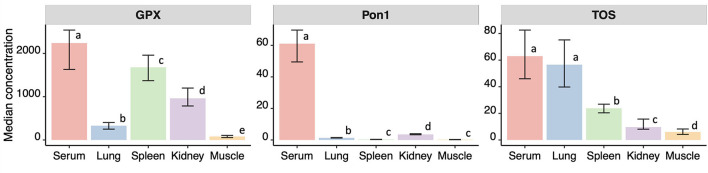
Median and confidence intervals of the median (in brackets) concentrations of p-nitrophenyl esterase activity (EA, in IU/ml), paraoxonase 1 (PON1, in IU/ml) in serum, glutathione peroxidase (GPX, in IU/L), and TOS (total oxidative status, in μmol/L) measured in serum, lung, spleen, kidney and muscle from 63 wild boars hunter-harvested in Toledo, Central Spain. Different letters indicate significant differences in pairwise comparisons at alpha = 0.05.

Among all the sample types, the highest concentration of GPX, PON1, and TOS was observed in boar serum. This difference was significantly higher in all the cases except when comparing TOS in serum and lungs (*p* > 0.05, permutation test). The patterns of biomarkers concentration among different organs and serum were specific for each biomarker (Figure 1).

### 3.2. Agreement between BOS measurements

There was a poor agreement among BOS measurements in boar serum and organs (Table 1). For example, the variation in the concentration of PON1 in serum differed between 118% in the lower range and 367% in the upper range compared to lung, spleen, kidney, and muscle. Similarly, GPX concentration in serum differed between 80% in the lower range and 1182% in the upper range compared to other tissues. In line with the previous results, the upper and lower limits of agreement for TOS concentrations were above 100%. The large confidence intervals in all three tissues hamper any attempt at an agreement with serum measurements.

**Table 1:** Descriptive statistics for the Bland Altman analysis of selected oxidative stress biomarkers: p-nitrophenyl esterase activity (EA)/paraxonase 1 (PON1), glutathione peroxidase (GPX), and total oxidative status (TOS) simultaneously measured in serum, lung, spleen, kidney, and muscle from 63 wild boar hunter-harvested in Toledo, Central Spain.

**Analyte**	**Sample pairs**	**Parameter**	**Unit (SD)**	**95% CI**	**Antilog**	**%**
EA/PON1	Serum-lung	Mean differences (bias)	0.27 (0.24)	(0.19–0.35)	1.86	–
		Upper limit	0.75	(0.61–0.89)	5.62	376
		Lower limit	−0.2	(−0.35 to 0.06)	0.63	123
	Serum-spleen	Mean differences (bias)	0.23 (0.26)	(0.14–0.32)	1.7	–
		Upper limit	0.73	(0.58–0.88)	5.37	367
		Lower limit	−0.28	(−0.43 to 0.13)	0.52	118
	Serum-kidney	Mean differences (bias)	0.48 (0.27)	(0.39–0.56)	3.02	–
		Upper limit	0.98	(0.83–1.13)	9.55	653
		Lower limit	−0.03	(−0.18 to 0.12)	0.93	209
	Serum-muscle	Mean differences (bias)	0.33 (0.32)	(0.22–0.43)	2.14	–
		Upper limit	0.95	(0.76–1.13)	8.91	677
		Lower limit	−0.29	(−0.48 to 0.11)	0.51	163
GPX	Serum-lung	Mean differences (bias)	0.51 (0.27)	(0.41–0.6)	3.24	–
		Upper limit	1.04	(0.88–1.2)	10.96	772
		Lower limit	−0.03	(−0.19 to 0.13)	0.93	231
	Serum-spleen	Mean differences (bias)	0.11 (0.21)	(0.04–0.19)	1.29	–
		Upper limit	0.53	(0.41–0.66)	3.39	210
		Lower limit	−0.31	(−0.43 to 0.18)	0.49	80
	Serum-kidney	Mean differences (bias)	0.35 (0.3)	(0.25–0.45)	2.24	–
		Upper limit	0.93	(0.76–1.11)	8.51	627
		Lower limit	−0.24	(−0.41 to 0.06)	0.58	166
	Serum-muscle	Mean differences (bias)	0.42 (0.38)	(0.29–0.54)	2.63	–
		Upper limit	1.16	(0.94–1.38)	14.45	1182
		Lower limit	−0.33	(−0.55 to 0.11)	0.47	216
TOS	Serum-lung	Mean differences (bias)	2.19 (49.84)	(−14.67 to 19.05)	–	–
		Upper limit	99.87	(70.78–128.96)	–	–
		Lower limit	−95.49	(−124.58 to 66.41)	–	–
	Serum-spleen	Mean differences (bias)	0.41 (0.28)	(0.32–0.51)	2.57	–
		Upper limit	0.96	(0.8–1.12)	9.12	655
		Lower limit	−0.13	(−0.3 to 0.03)	0.74	183
	Serum-kidney	Mean differences (bias)	53.12 (35.74)	(41.03–65.21)	–	–
		Upper limit	123.17	(102.31–144.02)	–	–
		Lower limit	−16.92	(−37.78 to 3.94)	–	–
	Serum-muscle	Mean differences (bias)	58 (35.12)	(46.12–69.89)	–	–
		Upper limit	126.84	(106.34–147.34)	–	–
		Lower limit	−10.84	(−31.34 to 9.66)	–	–

Mean differences indicate the average differences of each analyte recorded in each pair of biological samples (e.g., serum-lung). In the case of total agreement, this value should be equal to zero, so a positive value indicates that on average, the analyte in the first biological sample (e.g., serum) measures 2.19 units (e.g., TOS) more than in the second (e.g., lung). The standard deviation (SD), and the upper and lower limits at 95% CI (Bias-1.96 SD and Bias +1.96 SD) have also been calculated. When differences were not normally distributed, the raw data was log-transformed and the antilog back-transformation is shown in the table. Upper and lower limits of the agreement have also been shown in %.

### 3.3. Effect of TB on BOS in different tissues

With very few exceptions (i.e., PON1 in serum or GPX in spleen), the BOS concentrations were low in tissues from TB-infected individuals. Significant differences between healthy and diseased boars were only detected in the serum and muscle of individuals developing mild and severe TB disease status. In particular, p-nitrophenyl esterase activity (EA) in the muscle of healthy boar was between 1.8 times to 2.7 times higher than in individuals with mild (*t* = 2.41, *p*-value = 0.02) and severe (*t* = 3.7, *p*-value = 0.001) TB. Regarding GPX, statistically significant differences between healthy and TB-affected individuals were only detected in the muscle of severely affected boar (*t* = 3.41, *p*-value = 0.02). Finally, statistically significant differences in TOS concentrations were only observed in serum, particularly between healthy and mild TB-affected individuals (*t* = 2.92, *p*-value = 0.01). Mean and standard deviations of EA (PON1 in serum), GPX, and TOS concentrations in serum, lung, spleen, kidney, and muscle from healthy and sick boars are summarized in Table 2.

**Table 2:** Mean ± standard deviation for PON1 (paraoxonase 1), EA (p-nitrophenyl esterase activity), GPX (glutathione peroxidase), and TOS (total oxidative serum) measured in serum, lung, spleen, kidney, and muscle from healthy and TB-infected (mild and severely affected) wild boar, hunter-harvested in central Spain.

**Biological sample**	**Indicator**	**Healthy**	**Mild TB**	**Severe TB**
Serum	PON1	56.11 ± 23.81	60.6 ± 8.8	39.4 ± 13.4
	GPX	2564.0 ± 1847.83	2547.4 ± 282.6	1393.6 ± 331.3
	TOS	136.2 ± 36.1	69.2 ± 12.1^*^	81.1 ± 58.9
Lungs	EA	1.49 ± 0.11	1.43 ± 0.14	1.13 ± 0.31
	GPX	339.34 ± 24.9	341.89 ± 33.21	302.66 ± 46.54
	TOS	71.17 ± 11.41	68.49 ± 14.29	68.78 ± 26.93
Spleen	EA	0.37 ± 0.02	0.36 ± 0.03	0.42 ± 0.04
	GPX	1668.41 ± 134.05	1782.0 ± 191.23	1538 ± 252.31
	TOS	23.12 ± 2.45	28.51 ± 3.67	30.51 ± 5.58
Kidney	EA	4.51 ± 1.93	3.83 ± 1.67	5.24 ± 1.23
	GPX	1036.60 ± 142.88	1126.85 ± 150.71	841.61 ± 145.21
	TOS	12.41 ± 1.88	14.19 ± 3.13	12.8 ± 2.91
Muscle	EA	0.41 ± 0.09	0.22 ± 0.01^*^	0.15 ± 0.01 ^*^
	GPX	112.27 ± 14.54	91.37 ± 14.54	44.03 ± 11.62 ^*^
	TOS	8.24 ± 1.37	6.31 ± 1.22	4.85 ± 1.74

PON1, EA, and GPX have been measured in IU/L, and TOS in μmol/L. In all cases, we have used no hemolytic samples (n = 20 healthy, n = 16 mild-TB and n = 6 severe-TB). A *t*-test on 1000 bootstrapped replications has been used to compare the mean values of our ROS bioindicators between health and TB-affected boars. ^*^Indicates statistically significant differences at alpha = 0.05 after Bonferroni correction.

## 4. Discussion

We investigated the most reliable biological sample to assess OS in TB-infected wild boar. The BOS concentrations in serum were significantly higher than in other wild boar tissues such as lungs, spleen, kidney, or muscle. The poor agreement between BOS measures in different samples difficult the assessment of oxidative stress in the wild boar-TB systems. Only some tissues (e.g., TOS in serum and GPX or TOS in muscle) presented statistical differences in BOS concentrations between healthy and TB-affected individuals. These results highlight the importance of refining our understanding of intraindividual differences in OS assessments, particularly, when selecting appropriate samples for assessing specific animal populations' stressors.

Using OS biomarkers to evaluate chronic and acute stressors in wildlife needs to consider potential quantitative intraindividual differences among the biological samples collected. In our study, the higher levels of TOS, EA/ PON1, and GPX observed in serum compared to other tissues might be the additive result of BOS activity from different tissues transported and quantified in serum. More specifically, the activity of GPX varies depending on intra-individual characteristics like the tissues sampled (26, 52, 53), and time of sampling (54) and inter-individual factors like physiological status and sex (54, 55). A similar intra- and inter-individual variation have also been described in the activity of PON1 (42, 56, 57) and a variety of other BOS (24, 58). However, a higher BOS concentration in serum or other specific tissue does not necessarily imply a better sample for OS assessment. For instance, Gassó et al. (35) observed that the variability of OS was correlated with disease status in boars experimentally infected with TB. In the same work, and in line with our results, revealed that TB status was not the main factor explaining the observed variability in OS biomarkers, probably because other intrinsic [e.g., co-infections (33)] and extrinsic factors (e.g., food availability) may hide the TB effects. Our results highlight the need for baseline experiments to confirm the association between specific BOS and a stress source (e.g., disease) before their use as a proxy for animal health (23).

The poor agreement between BOS observed in our study hampers the opportunity of interchange markers between sample origins when assessing OS in wildlife. Variations between conspecifics in BOS at the species and individual scales (e.g., sex, age) are well-documented in different systems (59–61). However, intraindividual differences in BOS, like those observed in our study, have not received much attention despite their significant importance for an adequate sampling plan. This problem was partially addressed in a meta-analysis performed by Isaksson (60) to compare the influence of sampling methods [i.e., invasive sampling (e.g., liver, kidney, and lungs) and less-invasive sampling (blood and urine)] in the OS of vertebrates and invertebrates exposed to environmental pollution. Invasive sampling was more reliable in evaluating OS due to a smaller marker's concentration variation than less-invasive sampling. This result led to the hypothesis that in the case of contaminants and Eco toxicological studies, organs (e.g., the liver) are likely to be a better source of BOS due to their direct role in detoxifying harmful metabolites and pollutants. Tuberculosis in wild boar is characterized by causing a wide variety of pathophysiological consequences, including non-specific inflammatory responses and focalized gross lesions in lymph nodes, lungs, liver, mesenteric lymph nodes, and spleen (33, 34). These multiple potential OS sources might influence the agreement among biological samples highlighting the importance of context-specific consideration when assessing OS in wildlife populations. This includes context-specific extrinsic factors like species and stressor stimulus and context-specific intrinsic factors like biological samples and the physiological mechanisms of the species of interest to deal with the stressor stimulus.

Evidence of altered OS balance in wild boars infected with TB was detected only in animals with mild disease presentation. This can be explained in part due to the ability of wild boars to cope with TB infection (62), supporting the role of wild boar as a TB reservoir in the Mediterranean basin (30), and by the specific role of OS during early stages of TB development in the host. An increase in OS is one of the main physiological mechanisms described in humans and rats to prevent the multiplication of *Mycobacterium* spp. and TB-like gross lesions (63, 64). This mechanism is likely important in mild TB presentation, maintaining granulomatous lesions in a latent form and directly increasing the OS imbalance. In addition, Gassó et al. (35) reported a similar difficulty detecting altered OS in wild boar naturally infected with TB, hypothesizing that TB alone was not enough to trigger OS imbalance and that host intrinsic factors may also play an important role in OS levels. In this regard, special attention should be directed to the sex and age of the host as they have a direct influence on the immune and stress response of mammals (59–61).

## 5. Conclusions

The BOS evaluated in this study were detected in higher concentrations in serum compared to samples from other tissues. A poor agreement of BOS among samples was observed, making OS comparison among body fluids and tissue very difficult. In addition, organs from wild boars affected with mild TB (i.e., lung, spleen, kidney, muscle, and serum), were not in oxidative imbalance compared to healthy control animals. This suggests that wild boars may cope well with TB, developing an efficient antioxidant response to avoid OS. Our data indicate that more context-specific research on the association of BOS with stress factors like diseases is needed to allow the use of these parameters in field conservation programs.

## Data availability statement

The original contributions presented in the study are included in the article/supplementary material, further inquiries can be directed to the corresponding author/s.

## Author contributions

Study conception and design: DG, ES, and RC. Data collection: DG, AT, DR, PG, PF-L, GM, and WG. Analysis and interpretation of results: DG, AT, OA, RV, ES, RC, and GM. Drafted manuscript preparation: OA, DG, and ES. All authors reviewed the results and approved the final version of the manuscript.
